# Gain of the short arm of chromosome 2 (2p gain) has a significant role in drug‐resistant chronic lymphocytic leukemia

**DOI:** 10.1002/cam4.2123

**Published:** 2019-05-07

**Authors:** Fotini Kostopoulou, Clementine Gabillaud, Elise Chapiro, Beatrice Grange, Julie Tran, Simon Bouzy, Michael Degaud, Hussein Ghamlouch, Magali Le Garff‐Tavernier, Karim Maloum, Sylvain Choquet, Veronique Leblond, Jean Gabarre, Anne Lavaud, Veronique Morel, Damien Roos‐Weil, Madalina Uzunov, Romain Guieze, Olivier A. Bernard, Santos A. Susin, Olivier Tournilhac, Florence Nguyen‐Khac, the French Innovative Leukemia Organization (FILO) group

**Affiliations:** ^1^ Service d‘Hématologie Biologique Sorbonne Université Hôpital Pitié‐Salpêtrière APHP Paris France; ^2^ Molecular Diagnostics Laboratory KARYO Ltd Thessaloniki Greece; ^3^ INSERM U1138 Centre de Recherche des Cordeliers Sorbonne Université Paris France; ^4^ Gustave Roussy INSERM U1170 Université Paris‐Saclay Villejuif France; ^5^ Service d'Hématologie Clinique Sorbonne Université Hôpital Pitié‐Salpêtrière APHP Paris France; ^6^ Service d'Hématologie Clinique et de Thérapie Cellulaire Université Clermont Auvergne Clermont‐Ferrand France

**Keywords:** 2p gain, chronic lymphocytic leukemia, drug resistance

## Abstract

The different types of drug resistance encountered in chronic lymphocytic leukemia (CLL) cannot be fully accounted for by the 17p deletion (and/or *TP53* mutation), a complex karyotype (CK), immunoglobulin heavy‐chain variable region genes (*IGHV*) status and gene mutations. Hence, we sought to assess the associations between recurrent genomic abnormalities in CLL and the disease's development and outcome. To this end, we analyzed 64 samples from patients with CLL and gain of the short arm of chromosome 2 (2p+), which is frequent in late‐stage and relapsed/refractory CLL. We found that fludarabine/cyclophosphamide/rituximab (a common first‐line treatment in CLL) is not effective in removing the 2p+ clone ‐ even in samples lacking a CK, the 17p deletion or unmutated *IGHV*. Our results suggest strongly that patients with CLL should be screened for 2p+ (using karyotyping and fluorescence in situ hybridization) before a treatment option is chosen. Longer follow‐up is now required to evaluate bendamustine‐rituximab, ibrutinib, and idelalisib‐rituximab treatments.

## 1 INTRODUCTION

Chronic lymphocytic leukemia (CLL, the most common lymphoproliferative disorder among elderly adults in Western countries) is characterized by the progressive accumulation of mature CD5^+^ B lymphocytes in the bone marrow, peripheral blood, and secondary lymphoid organs. The prevalence of CLL increases with age, and its clinical profile ranges from CLLs that may not require treatment to others that progress aggressively.^1^ In addition to conventional immunochemotherapies (such as fludarabine‐cyclophosphamide‐rituximab [FCR] and bendamustine‐rituximab [BR]), a number of novel drugs have significantly modified the treatment strategies in this disease; they include kinase inhibitors (ibrutinib and idelalisib, targeting Bruton's tyrosine kinase and phosphatidyl‐inositol 3‐kinase, respectively), and an antagonist of the antiapoptotic Bcl‐2 protein (venetoclax).^2^

Genetic alterations (such as gains and losses of chromosomal regions) have prognostic value in CLL, due to their significant impact on the drug response and overall survival (OS).^1^, ^3^ Hence, these alterations should be taken into account when defining personalized treatments.^1^ The most frequent genetic abnormalities in CLL (deletions [del] of 11q, 13q, and 17p, and trisomy [tri] 12) have been extensively studied; del(17p) (which is tightly linked to *TP53* mutation) is associated with the worst prognosis.^3^, ^4^ Although del(17p) and/or a *TP53* mutations are found in up to 40% or 50% of patients with relapsing or refractory CLL, other genetic abnormalities may also be associated with drug resistances.^5^, ^6^ Several recurrent mutations have been described in CLL (in around 20% of patients) but cannot account for all the observed resistance.^7^ Lastly, a complex karyotype (CK) (observed in fewer than 20% of patients with CLL) might be associated with poor prognosis.^8^ It is therefore essential to understand the role of each recurrent genomic abnormality in the development and outcome of CLL, in order to assess individual risk factors. Furthermore, each genomic abnormality may create abnormal and potentially druggable activities.

We and others have previously reported that the gain of the short arm of chromosome 2 (2p+) is recurrent and frequent in late‐stage CLL (corresponding to about 15% of patients).^9^, ^10^, ^11^, ^12^ However, this abnormality is rarely analyzed specifically in clinical trials or in routine clinical practice. The 2p+ is associated with markers of a poor prognosis, such as 11q deletion and unmutated immunoglobulin heavy‐chain variable region gene (*IGHV*) status. Moreover, we have shown that the gene coding for exportin 1 (*XPO1*, located in 2p) has a central role in drug resistance in 2p+ CLL.^12^, ^13^ However, as the whole short arm of chromosome 2 is often gained in patients with 2p+ CLL, other known proto‐oncogenes located in the same region (such as *REL* and *MYCN*) might also be associated with CLL drug resistance.^9^, ^10^, ^11^, ^12^, ^13^

With a view to gaining a better understanding of prognostic and drug resistance factors in CLL, we have evaluated the role of 2p+ in disease progression and its response to treatment.

## 2 METHODS

### 2.1 Cases of CLL

We retrospectively collected data on 47 patients with 2p+ CLL, all of whom were diagnosed and/or treated at Pitié‐Salpêtrière Hospital (Paris, France) between 1990 and 2018. We also included 17 patients with 2p+ CLL who had been included and prospectively assessed by other centers as part of the ICLL1 BOMP trial (ClinicalTrials.gov identifier: NCT01612988).^5^ The diagnosis of CLL was based on lymphocytosis and immunophenotyping, according to the International Workshop on Chronic Lymphocytic Leukemia criteria.^1^ Patients with CLL were routinely examined for their *IGHV* status. Longitudinal cytogenetic analyses (ie at least two samples over time) were available for 26 of the 64 patients included in the study. In line with the ethical tenets of the Declaration of Helsinki, all the patients provided their informed consent to participation in the study. The study protocol was approved by the local investigational review board (*CPP Ile de France VI*, Paris, France) on 21 May 2014.

### 2.2 Karyotyping and fluorescence in situ hybridization

To obtain R‐banded chromosomes, cells from peripheral blood samples were stimulated with CpG‐oligonucleotides and interleukin‐2 (IL‐2) in culture for 72 hours, using standard techniques.^14^ All Ks were described according to the International System for Human Cytogenetic Nomenclature (ISCN) 2016. Complex Ks were defined as the presence of three or more numerical or structural chromosomal abnormalities, and highly complex Ks (HCKs) were defined as the presence of five or more abnormalities.^15^

Fluorescence in situ hybridization (FISH) was performed on metaphase and interphase nuclei, using the following panel of probes: MYCN (Abbott, Rungis, France), TP53/ATM (Cytocell Ltd, UK), LSI D13S319/12cen (Metasystems, Altlusshein, Germany), and “home‐grown” bacterial artificial chromosome probes for the *XPO1* (RP11‐240F4+RP11‐477N2), *REL* (RP11‐373L24) and *BIRC3* (RP11‐177O8) genes were selected using the University of California Santa Cruz Genome Bioinformatic database (NCBI37/hg19 build) and obtained from Genoscope (Evry, France). Results were recorded using a fluorescent microscope (Olympus) with appropriate filters, and Isis imaging software (Metasystems, Heidelberg, Germany). All FISH preparations were scored by two independent assessors, with at least two independent counts of 100 nuclei/probe/assessor.

### 2.3 Single nucleotide polymorphism array analysis

Single nucleotide polymorphism (SNP) array analyses were performed as described previously.^13^

### 2.4 Mutations in *TP53*

For patients with more than one sample, *TP53* mutations were analyzed using: (a) Sanger sequencing of exons 4‐10 (n = 2); (b) next‐generation sequencing on a MiSeq^®^ system (Illumina, San Diego, CA) using the CLL MASTR PLUS kit (Agilent, Santa Clara, CA) (n = 6); or (c) as previously described in the report on BOMP trial (n = 11).^5^

### 2.5 Statistical analysis

The time to first treatment (TTFT) was defined as the time interval between diagnosis and first‐line treatment. The OS time was defined as the time interval between diagnosis and death or (in the absence of death) last follow‐up. OS was analyzed using the Kaplan‐Meier method. The log‐rank test was used for intergroup comparisons of TTFT or OS curve. The variables analyzed were CK, HCK, del(13q), del(11q), *IGHV* mutation status, *XPO1*/*REL*/*MYCN* gain (homogeneous gain vs heterogeneous gain). Quantitative variables were reported as the median (range) or the median [95% confidence interval (CI)], and categorical variables were reported as the number (percentage).

## 3 RESULTS

### 3.1 Characteristics of the study population

A total of 64 patients with 2p+ CLL were included in the study. Most of the patients were male (51 out of 64, 80%), and the median (range) age at diagnosis was 60 (42‐78) (Table S1). Of the 63 patients with available data, 41 (65%) had not been treated before karyotyping; the median (range) time between diagnosis and karyotyping was 5 months (0‐88). The Binet stage was known for 36 of these 41 untreated patients, with four (11%) stage A cases, 27 (75%) stage B cases, and five (14%) stage C cases. The *IGHV* status was unmutated in 50 of the 56 tested patients (89%).

The median (range) number of lines of treatment was two (0‐8). Twenty‐eight of the 64 patients (44%) died during the study period, with a median follow‐up from diagnosis of 79 months (0‐317). At last follow‐up, 61 of the 62 patients with available data (98%) had been treated (Table S1).

**Figure 1:**
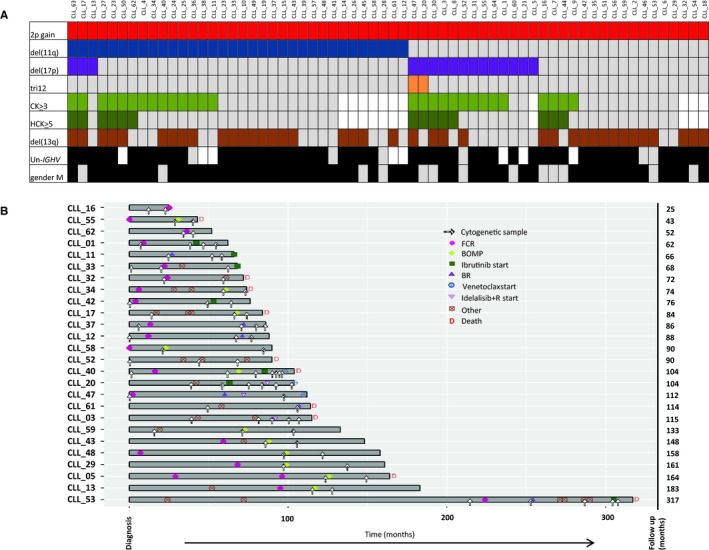
A, Distribution of chromosomal abnormalities in 64 patients with 2p+ CLL. Each column represents a patient, and each row represents a parameter. Color code: gray, absence; black or other colors, presence; white, not available. CK: complex karyotype, defined as three or more chromosomal abnormalities; HCK: highly complex karyotype, defined as five or more chromosomal abnormalities; Un‐*IGHV*: unmutated *IGHV*. B, Description of longitudinal samples for 26 patients with 2p+ CLL. FCR: fludarabine/cyclophosphamide/rituximab; BR: bendamustine‐rituximab; BOMP: bendamustine/ofatumumab and methylprednisolone

All 64 patients had a 2p+ (as evidenced by FISH and/or SNP array analysis), and 53 of them had been successfully karyotyped. Seven of these 53 patients (13%) had a normal karyotype (K), and 28 (53%) had a CK, including 14 (26%) HCKs. The results were similar when we considered only the 35 patients who had not been treated before karyotyping (Figure 1A, Table S1, and Figure S1).

The 2p+ could be clearly identified by R‐banding in 17 of the 53 (32%) Ks (Table S2); it was never the sole abnormality, and was present in the main clone in ten of the 17 cases (59%), in a subclone in four cases (24%), and in an independent clone in three cases (18%). It is noteworthy than when the 2p+ was subclonal, it occurred after 11q deletion. The gain of 2p material was due to a duplication in the short arm of chromosome 2 in four of the 17 cases (24%), while an isochromosome 2p was present in one case. In the remaining cases, 2p was attached to different chromosomes (chromosomes 8, 11, 18, 20, and 22). The most frequent partner chromosome was chromosome 18 (five out of 17, 29%) (Tables S1 and S2).

Using FISH and/or an SNP array, del(13q) was identified in 39 of the 64 patients (61%), del(11q) in 34 (53%), del(17p) in 16 (25%), and tri12 in two (3%). When considering only the patients who had not been treated before analysis, the results were quite similar (Figure 1A, and Tables S1 and S2).

To investigate the three main genes located on the short arm of chromosome 2 (*MYCN* [2p24], *REL* [2p16] and *XPO1* [2p15]), we performed a locus‐specific FISH analysis of the 48 patients with available material (Table S2). The median (range) percentage of cells with 2p+ was 40% (8%‐96%). In 42 of the 47 cases (89%), there were three copies of the gained genes. In five cases, we observed a mixture of cells with three, four, or five copies. Interestingly, we found discrepancies between the three gene loci. To rule out variations due to the technical limitations of FISH, we considered that a difference of more than 20% in the 2p+ cell count was a marked change—unless a gain was not detected for one gene or if the same differences were observed in the longitudinal analysis (Figure S2A). Most cases (38 out of 48, 79%) had a homogeneous gain of the three genes, whereas the two regions (2p15‐16 and 2p24) were not gained in the same manner in the remaining ten patients (21%). In the latter cases, the percentages of cells with *XPO1* and *REL* gains were always correlated, whereas the percentage of cells with an *MYCN* gain differed. More specifically, chromosomal region 2p15‐16 (*XPO1/REL*) was gained in a higher proportion of tumor cells than the 2p24 region (*MYCN*) in eight of the 48 patients (17%). In five of these eight patients, a gain of the *MYCN* gene was not detected at all. Only two (4%) patients had a higher percentage of cells having gained the 2p24 region (including *MYCN* gene) than cells having gained the region 2p15‐16 (including the *XPO1* and *REL* genes).

Given that *BIRC3* was recently reported to be involved in the 11q deletion, we performed FISH using two specific probes (encompassing either *ATM* or *BIRC3*) for 50 patients.^16^ We found that 24 of the 50 cases (48%) had an *ATM* deletion, and 20 of these 24 cases (83%) had a *BIRC3* deletion. In four of the 24 cases (25%), we observed the deletion of *ATM* alone, while none of the patients had a *BIRC3* deletion alone. The percentages of cells with a deletion were similar for both genes in all cases. The median (range) percentage of cells with a deletion was 71% (11%‐99%) (Figure S2B).

### 3.2 Time to first treatment and overall survival

The median TTFT was 16 months; the median OS time was 124 months (Table S1). In a univariate analysis, the only parameter associated with significantly shorter TTFT was the del(17p) (2.5 vs 22 months in 2p+ CLL with or without del(17p), respectively; *P *=* *.04). There were no statistically significant differences in the impact on OS for all the analyzed parameters (Table S3).

### 3.3 Patients with more than one cytogenetic analysis (n = 26)

We were able to perform a longitudinal cytogenetic analysis for 26 patients with 2p+ CLL (Figure 1B). All 26 had received treatment, with a median (range) follow‐up time of 90 months (25‐317). The median [95% CI] TTFT was 23.5 months [12‐43]. Ten of the 26 CLL (38%) patients with 2p+ died during the study period; the median [95% CI] OS time was 164 months [114‐not reached]. For the 17 patients with available data, the 2p+ was observed before treatment in 13 (77%) cases and after treatment in four cases (23%). It is noteworthy that none of the 15 patients lacking a 17p deletion had a *TP53* mutation.

We next analyzed the change in the 2p+ clone and recurrent chromosomal abnormalities in patients treated with a panel of drugs (Table 1). To exclude variations related to the technical limitations of FISH, we considered that a difference of more than 20% in the number of 2p+ cells was a marked change between pretreatment and relapse, except when a clone was no longer detected or when we were able to perform a valid comparison with other clones. The proportion of cells with *MYCN/REL/XPO1* gains remained stable in all but one patient (CLL_58), in whom a *MYCN* gain was detected at relapse (*XPO1* and *REL* had been gained before treatment). The median (range) number of lines of treatment was two (1‐8), and the patients were assessed in detail as a function of the drugs included in each treatment regimen.

**Table 1:** Change over time in the 2p+ clone after various treatments

Treatment	Prior lines of treatment	2p+ before treatment	CK (>3) before treatment	del(17p) before treatment	*TP53* mut^d^ before treatment	del(11q) before treatment	Un‐*IGHV*	Time to next 2p+ evaluation	Disease status when the second sample was collected	Decrease in 2p+	Stable 2p+	Increase in 2p+
**Patients not treated when 2p+ was first detected**												
FCR n = 5	0	5/5	1/3	0/5	0/3	3/5	4/4	Median (range): 45 m (32 m‐59 m)	relapse	0	3	2
BR n = 1	0	1/1	1/1	0/1	0/1	1/1	na	26 m	relapse	0	1	0
**Patients already treated when 2p+ was first detected**												
FCR n = 2	0‐1	1/2	0/1	0/2	0/1	1/2	0/1	26 m‐29 m	relapse	0	1	1^a^
BR n = 2	1	2/2	1/2	0/2	0/2	2/2	1/1	3 m‐7 m	remission	2	0	0
BOMP n = 11	1‐3	10/11	4/10	5/11	5/11	8/11	11/11	Median (range): 14 m (5 m‐62 m)	relapse	4^b^	4	3^c^
Ibrutinib n = 5	1‐6	5/5	3/5	3/5	2/3	1/5	3/4	Median (range): 11 m (5 m‐20 m)	normal/decreased lymphocytosis (n = 4)/relapse (n = 1)	2	3	0
Idelalisib + R n = 2	2	2/2	2/2	2/2	na	0/2	2/2	13‐16 m	partial remission (n = 1)/relapse (n = 1)	0	2	0

CK: complex karyotype > 3 chromosomal abnormalities; na: not available; Un, unmutated.

a The 2p gain appeared after FCR treatment (CLL_1).

b In one case (CLL_43), the 2p gain was no longer detected.

c In one case (CLL_5), the 2p gain appeared after BOMP treatment.

d No cases with a *TP53* mutation (mut) but no del(17p).

### 3.4 Longitudinal analysis after first‐line immunochemotherapy

#### 3.4.1 Fludarabine/cyclophosphamide/rituximab

Sixteen of the 19 patients treated with FCR in the longitudinal subgroup relapsed, after a median (range) time interval between the end of treatment and relapse of 24 months (5.4‐78); the other three patients have not finished their course of treatment. Cytogenetic data before front line FCR and at relapse (evaluation pre‐next treatment) were available for six patients (Table 1).

**Figure 2:**
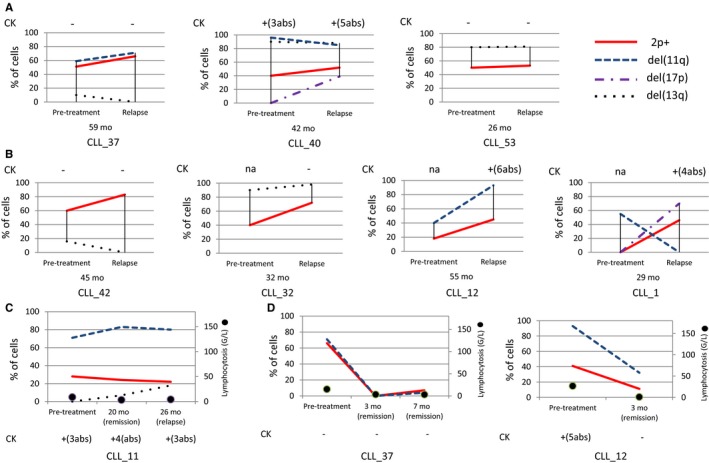
Treatment of 2p+ CLL with FCR (n = 7) (A, B) or BR (n = 3) (C, D). A and B, Samples before treatment and at relapse (ie, the evaluation before the next treatment). Before relapse, all the patients achieved complete remission after FCR. With the exception of one patient (CLL_53), FCR was the first‐line treatment. The time interval between the first day of treatment and the cytogenetic analysis at relapse is indicated below each graph. A, The 2p+ clone remained stable at relapse after FCR therapy. Patient CLL_53 underwent a splenectomy and received chlorambucil before FCR. B, After FCR therapy, the 2p+ clone increased at relapse. In CLL_1, the 2p+ clone appeared after FCR treatment. After FCR treatment, none of the patients displayed a reduction of the size of the 2p+ clone at relapse. C, After first‐line treatment with BR, the 2p+ was stable at relapse. D, After second‐line treatment with BR. In patient CLL_37, the abnormal clones were transiently negative for 2p+. The frequency of the 2p+ clone decreased but was still detected in the two patients in complete remission. Abs: abnormalities; CK: complex karyotype

In two patients (CLL_37 and CLL_40), the percentage of 2p+ cells was stable at relapse. Furthermore, the clone del(13q) remained large and stable in one patient (CLL_40) but could no longer be detected in the other (CLL_37). The clone del(11q) also remained large and stable in both patients; the deletion encompassed *ATM* and *BIRC3* in one patient (CLL_37), and *ATM* only in the other (CLL_40). In patient CLL_40, del(17p) was detected after FCR treatment, along with a HCK (Figure 2A).

In four patients, the number of 2p+ cells had increased (by a factor of 1.5‐2) at relapse. The del(13q) remained stable in one of these patients (CLL_32) but disappeared in a second (CLL_42) (Figure 2B). Two patients (CLL_12, CLL_1) had a clone with del(11q), including both *ATM* and *BIRC3*. In patient CLL_12, the frequency of 2p+ and del(11q) had doubled after FCR treatment. Interestingly, del(11q) was detected at diagnosis in patient CLL_1; the 2p+ appeared after treatment (along with del(17p) and CK) but the del(11q) disappeared (Figure 2B).

#### 3.4.2 Bendamustine‐rituximab

One patient received first‐line treatment with BR. After clinical remission, he relapsed at 26 months; the clones 2p+ and del(11q) were stable but a del(13q) clone appeared (Table 1 and Figure 2C).

### 3.5 Longitudinal analysis after post‐relapse immunochemotherapy

#### 3.5.1 Fludarabine/cyclophosphamide/rituximab

One patient (CLL_53) underwent splenectomy, and received chlorambucil and then FCR. The percentage of cells with 2p+ and del(13q) remained stable at the time of relapse (Table 1 and Figure 2A). Overall, after FCR treatment, none of the patients displayed a reduction in the 2p+ clone at relapse (Table 1 and Figure 2A,B).

#### 3.5.2 Bendamustine‐rituximab

Two patients with available cytogenetic data were treated with BR after one course of FCR (Table 1 and Figure 2D). They were both in clinical remission after 3 and 7 months, although persistent clonal abnormalities were still present in peripheral blood cells including 2p+ (even though the abnormal clones were transiently negative in patient CLL_37).

#### 3.5.3 Bendamustine/ofatumumab and methylprednisolone (BOMP)

**Figure 3:**
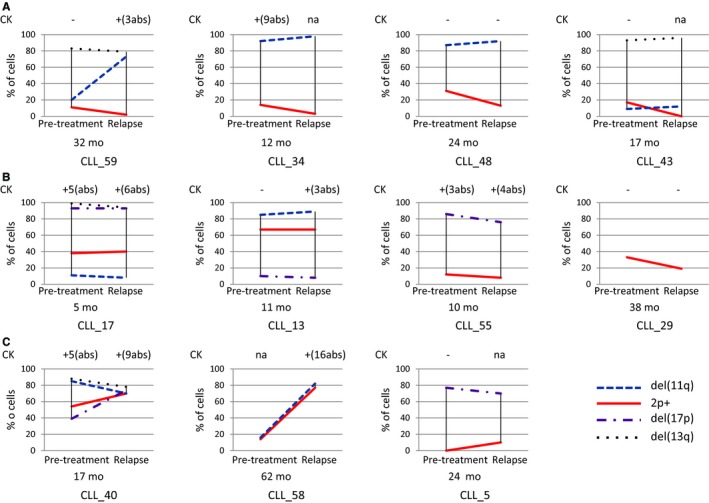
Treatment of patients with 2p+ CLL (n = 11) with BOMP. Only two patients (CLL_59 and CLL_29) were in complete remission after the BOMP therapy. A, At relapse, the size of the 2p+ clone had decreased in four patients, including one patient with no detectable 2p+ cells (CLL_43). The percentage of 2p+ cells was below 5% in two patients (CLL_59, CLL_34). B, The 2p+ clone was stable in four patients. C, The size of the 2p+ clone increased in three patients, including one patient with a detectable clone at relapse only (CLL_5). Abs: abnormalities; CK: complex karyotype

A total of 17 of the 64 patients were included in the BOMP trial, including one patient with a detectable 2p+ clone at relapse. All of them relapsed, with a median (range) time from inclusion to relapse of 13 months (5.4‐38.2). Cytogenetic data before BOMP treatment and at relapse (at an evaluation prior to selection of the following treatment) were available for 11 patients (Table 1). At relapse after the course of BOMP, the 2p+ decreased or disappeared in four patients (Figure 3A). It is noteworthy that the percentage of 2p+ cells was below 5% in two patients. The percentage remained stable in four other patients (Figure 3B). In three cases, BOMP therapy resulted in an increase in the 2p+. One of the three (CLL_5) did not display the clone before BOMP treatment (Figure 3C). Most FISH abnormalities were stable at relapse (four out of four del[13q], four out of five del[17p], and six out of eight del[11q]). Five of the eight cases with del(11q) included *ATM* and *BIRC3*. Of the two cases (CLL_59, CLL_58) with an elevated del(11q) clone at relapse, the deletion involved *ATM* alone in CLL_59 only. The number of karyotype abnormalities increased in five of the seven patients with available data.

### 3.6 Longitudinal analysis after post‐relapse treatment with a kinase inhibitor

#### 3.6.1 Ibrutinib

**Figure 4:**
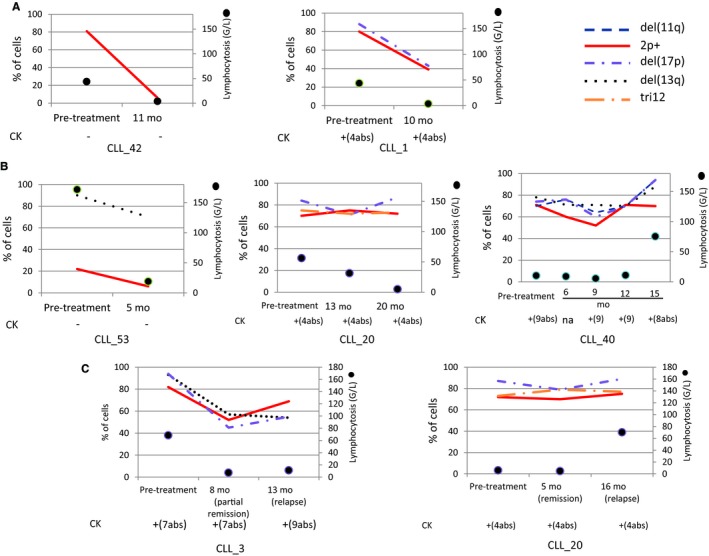
2p+ CLL treated with ibrutinib (n = 5) (A, B) and idelalisib‐rituximab (n = 2) (C). A, The size of the 2p+ clone decreased but was still detectable in patients in complete remission (CLL_42, CLL_1) after ibrutinib treatment. B, With ibrutinib, the size of the 2p+ clone was stable during follow‐up, even for a patient in complete remission (CLL_20). C, With idelalisib + R, the size of the 2p+ clone was stable at relapse for both patients. Abs: abnormalities; CK: complex karyotype

Five patients with available cytogenetic data received ibrutinib, after 1‐6 other lines of treatment (Table 1). After 11 and 10 months, CLL_42 and CLL_1 were in clinical remission, and showed a reduction (according to FISH) in the percentage of cells with 2p+ (Figure 4A). The percentage of cells with 2p+ remained stable in the other patients (as evidenced by karyotyping and FISH) after five (CLL_53), 20 (CLL_20), and 15 (CLL_40) months (Figure 4B). The patient CLL_40 relapsed at 15 months, and the patient CLL_53 died 6 months after the last cytogenetic analysis. The clone del(17p) fell by 50% in one patient (CLL_1) and remained stable in the other cases (CLL_20, CLL_40). The del(11q) involving *ATM* only persisted in CLL_40, as was also the case during the other treatments (FCR and BOMP). It is noteworthy that even though three patients were in clinical remission (CLL_42, CLL_1, and CLL_20), the abnormalities were still present in karyotyping and FISH analysis.

#### 3.6.2 Idelalisib‐rituximab (idelalisib‐R)

Two patients with available cytogenetic data were treated with idelalisib‐R after two other lines of treatment (Table 1). In patient CLL_3, lymphocytosis increased (up to 62 G/l) after a partial remission, and the patient died 4 months after the last cytogenetic analysis (9 years after diagnosis). Patient CLL_20 relapsed after 16 months. For both patients, the 2p+ clone was stable at relapse (Figure 4C).

Lastly, regarding the clonal outcome of the del(11q), the different treatment groups did not differ in terms of whether del(11q) encompassed *ATM* alone or both *ATM* and *BIRC3* (Table S4). The size of the deletion (whether encompassing one or two genes) remained stable during the course of the disease.

## 4 DISCUSSION

Here, we presented a detailed cytogenetic analysis of what is (to the best of our knowledge) the largest yet cohort of patients with 2p+ CLL (n = 64). A longitudinal analysis was possible in 26 cases (ie patients with more than one sample collected over the course of the disease). Our first key observation was that the male‐to‐female ratio (3.9:1) is higher than the one reported for CLL in general (1.5‐2:1).^17^ We therefore confirmed Jarosova et al.'s report of male predominance in 2p+ CLL.^18^ We also corroborated our previous report whereby 2p gain is frequent in untreated, late‐stage CLL (32 of the 36 cases [89%] of Binet stage B/C CLL in the present cohort) and in relapsed/refractory CLL (22% of the BOMP trial, unpublished data).^12^, ^13^ The 2p gain was associated with a CK in 53% of cases and a HCK in 26%; these percentages are higher than the literature values for CLL in general (15%‐18% and 4%, respectively).^8^, ^19^ In line with literature reports, we further confirmed that 2p gain is closely associated with del(11q) (53% of untreated patients in our series), unmutated *IGHV* status (in 89%) and—to a lesser extent—del(17p) (in 19%).^7^, ^12^, ^13^, ^20^ Conversely, tri12 was very rare in our patients with 2p+ CLL (3% here, vs 15% in CLL in general).^3^, ^8^ Interestingly, in contrast to CLL in general, we did not observe—in our cohort of 2p+ CLL—a significant association between the OS and the common poor prognostic factors, that is, del(17p), unmutated *IGHV* and HCK.^1^, ^15^ Lastly, 2p gain was overlooked by karyotyping in 68% of our cases; we therefore strongly recommend systematic FISH screening for this abnormality.

Rossi *et al*.^16^ reported the involvement of *ATM* and *BIRC3* in 11q deletion. As the del(11q) was present in more than a half of our cohort of 2p+ CLL cases, we investigated the two genes. We found that del(11q) involves both genes in the majority of cases, and that *BIRC3* is never deleted alone (as also shown by another group^21^). Moreover, whether del(11q) encompassed one or two genes did not seem to influence the treatment response in our cohort.

As 2p gain often involves the whole short arm of chromosome 2, we decided to study two previously described minimal regions of gain: 2p24 and 2p15‐16.^10^, ^12^, ^13^, ^18^, ^20^, ^22^ The 2p24 region encompasses *MYCN*, and the 2p15‐16 encompasses *XPO1* and *REL*, all of which are overexpressed in 2p+ CLL.^13^ *MYCN* and *REL* are already known oncogenes involved in lymphoid malignancies. *MYCN* (a member of the *MYC* family) is associated with a variety of tumors, including neuroblastoma.^23^ *REL* encodes a member of the NF‐Κb family of transcription factors involved in cell growth and differentiation, and is overexpressed in Hodgkin's lymphoma.^24^ Lastly, we recently highlighted the role of *XPO1* (coding for exportin 1) in the drug resistance associated with 2p+.^13^ In the majority of cases in our series, the three oncogenes had been gained together in the same proportions—thus confirming their relevance in CLL drug resistance. Nevertheless, we found that the two regions can also be gained differently; in some cases, *MYCN* is not gained. We therefore recommend investigating these two regions using FISH. As *MYCN* is never gained alone, at least one probe encompassing *REL* or *XPO1* should be used.

To evaluate clonal changes in patients with 2p+ CLL, we assessed the consequences of different drug treatments on 2p gain and other abnormalities. Nineteen patients were treated with FCR; this remains a major first‐line option for many patients with CLL, and is considered to be effective in cases with favorable features (including mutated *IGHV* status).^2^ We found that FCR is not able to eliminate the 2p clone. The majority of the FCR‐treated patients with 2p+ CLL relapsed within 3 years. Indeed, in our series, 2p gain increased or remained stable at relapse after FCR treatment. It even appears that FCR induced 2p gain in one patient. It is noteworthy that FCR was able to eliminate two clones with del(13q) and one with del(11q). Taken as a whole, our data suggest that the first‐line use of FCR is not a good choice for 2p+ CLL—even for patients lacking factors for a poor prognosis (*IGHV*‐U, del(17p), del(11q), and CK).

Three patients received first‐ or second‐line treatment with BR. Even a short period without a 2p+ clone was observed for one patient, the clone was still detected in all the patients in clinical remission. One patient (with first‐line BR) relapsed at 26 months but more follow‐up is required for the other two patients. Hence, we are not yet able to draw conclusions about BR and 2p+ CLL.

Eleven patients received BOMP therapy. This regimen included the fully humanized anti‐CD‐20 antibody ofatumumab, which is considered to be feasible and effective in patients with relapsed/refractory CLL ‐ including those with high‐risk clinical and laboratory features.^5^ All 11 patients had already received other treatments, and had factors linked to a poor prognosis. However, when looking specifically at the 2p clone, the frequency decreased in three patients or was even no longer detected in a fourth patient at relapse. In contrast, no other FISH abnormalities were eliminated, and the two del(11q) clones and the del(17p) clone increased. Our results suggest that BOMP is a good treatment option for some patients with 2p+CLL.

A number of new treatment options have recently emerged for patients with CLL, including inhibitors of Bruton's tyrosine kinase (ibrutinib) and phosphatidyl‐inositol 3‐kinase (idelalisib).^2^ In our study, five patients had been treated with ibrutinib. We found that the 2p gain clone either remained stable or decreased but was still detectable—even when the lymphocyte count had normalized. After treatment with idelalisib, the 2p gain was still detected in patients in clinical remission. It is noteworthy that all the other abnormalities were still present. Hence, longer follow‐up is required to evaluate these drugs’ long‐term impact on 2p+ CLL. Nevertheless, our initial data appear to indicate that ibrutinib or idelalisib do not eliminate the 2p+ clone. Three patients had started treatment with venetoclax.

In conclusion, the results of our large study indicate that 2p gain is frequent in relapsed/refractory CLL. The 2p gain is also associated with male gender, a HCK, del(11q), del(17p), and unmutated *IGHV* status. The gain can appear during natural disease progression or after treatment. The commonly used first‐line treatment FCR is not effective with regard to the 2p+ clone, even when a CK or del(17p) is absent. Treatment with BOMP appears to have an effect on the 2p+ clone in some patients. We need more follow‐up data for ibrutinib, idelalisib‐R, and bendamustine‐R. Overall, our results strongly suggest that it is essential to systematically screen for 2p gain (using karyotyping and FISH) to better characterize CLL.
