# Supplementary figures and images for: Gain of the short arm of chromosome 2 (2p gain) has a significant role in drug‐resistant chronic lymphocytic leukemia
Source: Cancer Med. 2019 May 7;8(6):3131–41. doi: 10.1002/cam4.2123 (PMC6558483; doi:10.1002/cam4.2123)

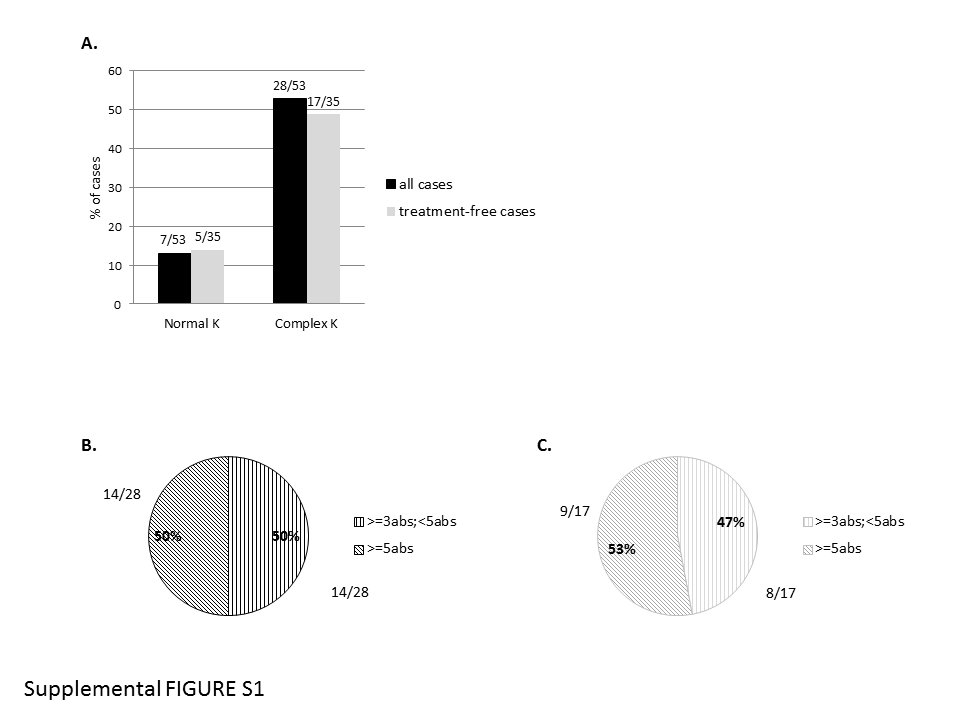

Supplement: Supplementary file 1 [file CAM4-8-3131-s001.TIF]

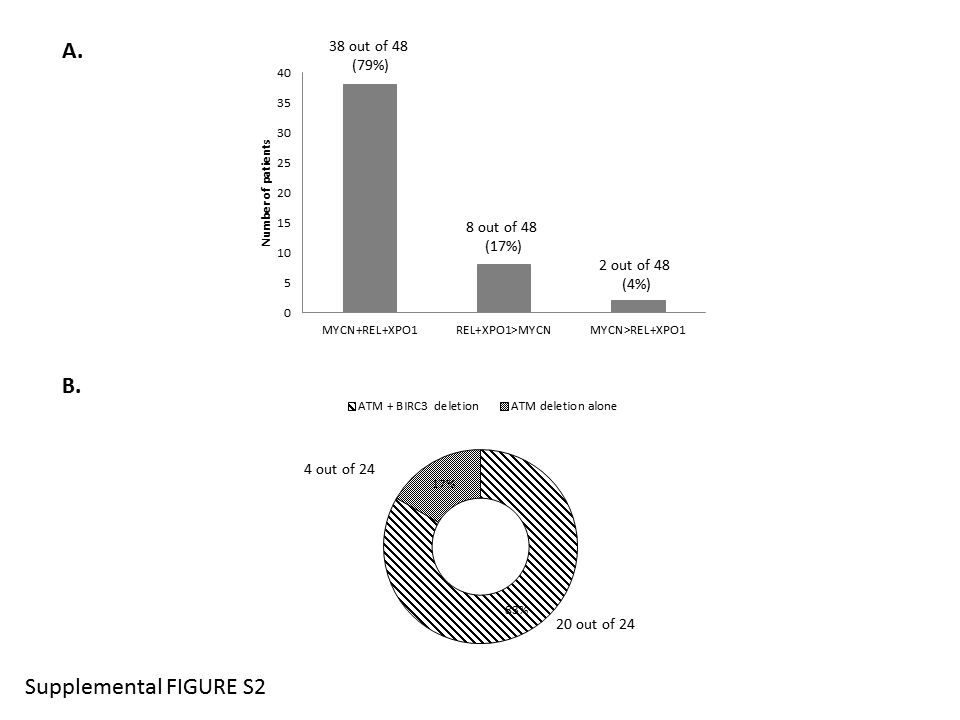

Supplement: Supplementary file 2 [file CAM4-8-3131-s002.TIF]
