# Supplementary material for: Gain of the short arm of chromosome 2 (2p gain) has a significant role in drug‐resistant chronic lymphocytic leukemia
Source: Cancer Med. 2019 May 7;8(6):3131–41. doi: 10.1002/cam4.2123 (PMC6558483; doi:10.1002/cam4.2123)
Supplement: Supplementary file 3 [file CAM4-8-3131-s003.docx]

| **Gender M** | 51/64 (80%) |
| --- | --- |
| **Age at diagnosis, median (range)** | 60 (42-78) |
| **Cytogenetic at time first detection of 2p+**  **..Karyotyping** (n=53*)  **Normal karyotype**  **Complex karyotype (>3 abnormalities)**  **Highly complex karyotype (>5 abnormalities)** | 7 (13%)  28 (53%)  14 (26%) |
| **..Recurrent 2p gain identified by karyotyping** (n=17**)  **…..t(2;18)**  **…..dup(2p)**  **…..t(2;20)**  **…..t(2;22)** | 5 (29%)  4 (24%)  2 (12%)  2 (12%) |
| **..FISH/SNP array** (n=64)  **del(13q)**  **del(11q)**  **del(17p)**  **tri12** | 39 (61%)  34 (53%)  16 (25%)  2 (3%) |
| **Unmutated *IGHV*** (n=56¤) | 50 (89%) |
| **Description of the population at last follow-up**  **…..Time from diagnosis to first treatment, median [95%CI]** (n=62¤)  **…..Number of lines of treatment, median** **(range)** (n=62¤)  **…..Deaths** (n=64¤)  **…..Overall survival, median [95%CI]** (n=60¤)  **…..Follow up from diagnosis, median (range)** (n=60¤) | 16 m [8-25]  2 (0-8)  28 (44%)  124m [114-not reached]  79 m (0-317) |
| **Patients not treated when 2p+ was first detected** (n=41¤)  **..Binet stage at cytogenetic study** (n=36¤)  **…..A**  **…..B**  **…..C** | 4 (11%)  27 (75%)  5 (14%) |
| **..Karyotyping** (n=35*)  **Normal karyotype**  **Complex karyotype (>3 abnormalities)**  **Highly complex karyotype (>5 abnormalities)** | 5 (14%)  17 (49%)  9 (26%) |
| **..FISH/SNP array** (n=41)  **…..del(13q)**  **…..del(11q)**  **…..del(17p)**  **…..tri12** | 26 (63%)  24 (58%)  8 (19%)  1 (2%) |
| **..Unmutated *IGHV*** (n=36¤) | 32 (89%) |
| **Patients treated when 2p+ was first detected** (n=22¤) |  |
| **..Number of previous lines, median (range)** | 3 (2-8) |
| …..chlorambucil  **…..**F/FC  **…..**FCR  **…..**alemtuzumab  **…..**bendamustine, ofatumumab, methylprednisolone  **…..**BR  **…..**idelalisib-R  **…..**Other | 3  4  14  5  1  1  1  4 |

**SUPPLEMENTAL TABLE S1.** Clinical and Cytogenetic data of 64 2p+ CLL

*Successful karyotyping

**The 2p gain was clearly identified by karyotyping in 17 patients. Some 2p abnormalities were recurrent.

¤ Patients with available data

m=months, chlorambucil, F: fludarabine, C: cyclophosphamide, B: bendamustine, R: rituximab
