# Supplementary material for: Gain of the short arm of chromosome 2 (2p gain) has a significant role in drug‐resistant chronic lymphocytic leukemia
Source: Cancer Med. 2019 May 7;8(6):3131–41. doi: 10.1002/cam4.2123 (PMC6558483; doi:10.1002/cam4.2123)
Supplement: Supplementary file 5 [file CAM4-8-3131-s005.docx]

| **Variable** |  | n | median [95%CI]  months | p-value |
| --- | --- | --- | --- | --- |
| **TTFT** |  |  |  |  |
| CK | Yes  No | 23  24 | 22 [6-41]  8.5 [6-25] | ns |
| HCK | Yes  No | 11  36 | 25 [2-not reached]  13.5 [7-not reached] | ns |
| *IGHV* unmutated | Yes  No | 46  4 | 16 [6-25]  29.5 [2-not reached] | ns |
| del(13q) | Yes  No | 32  25 | 16 [8-41]  16 [6-31] | ns |
| del(11q) | Yes  No | 29  28 | 16 [8-43]  15 [2-30] | ns |
| del(17p) | Yes  No | 16  41 | 2.5[1-41]  22[11-31] | **0.04** |
| *MYCN* gain | Yes  No | 36  5 | 22 [9-33]  23 [19-not reached] | ns |
| **OS** |  |  |  |  |
| CK | Yes  No | 25  25 | 104 [73-not reached]  317 [115-not reached] | ns |
| HCK | Yes  No | 11  39 | 124 [115-not reached]  115 [88-not reached] | ns |
| *IGHV* unmutated | Yes  No | 47  6 | 124 [104-not reached]  153 [90-not reached] | ns |
| del(13q) | Yes  No | 35  25 | 124 [114-not reached]  153 [64-not reached] | ns |
| del(11q) | Yes  No | 31  29 | 153 [114-not reached]  115 [72-not reached] | ns |
| del(17p) | Yes  No | 16  44 | 90 [68-not reached]  153 [115-not reached] | ns |
| *MYCN* gain | Yes  No | 39  5 | 115 [104-not reached]  Not reached | ns |

**SUPPLEMENTAL TABLE S3.** Univariate analysis of clinical and biological parameters on time to first treatment (TTFT) and overall survival (OS). CI: confidence interval, ns: not significant, n: number of 2p+ CLL, CK: complex karyotype, HCK: highly complex karyotype
