# Supplementary material for: Gain of the short arm of chromosome 2 (2p gain) has a significant role in drug‐resistant chronic lymphocytic leukemia
Source: Cancer Med. 2019 May 7;8(6):3131–41. doi: 10.1002/cam4.2123 (PMC6558483; doi:10.1002/cam4.2123)
Supplement: Supplementary file 6 [file CAM4-8-3131-s006.docx]

| Treatment | n | del*ATM* | del*BIRC3* | Decrease in del(11q) | Stable del(11q) | Increase in del(11q) |
| --- | --- | --- | --- | --- | --- | --- |
| FCR (n=4)  (first-line) | 2  1  1 | 2  1  1 | 1  1  1 | 1* | 2 | 1 |
| BR (n=3)  (first-line)  (after 1 line) | 1  2 | 1  2 | 1  2 | 2 | 1 |  |
| BOMP (n=8)  (R/R after 1 to 3 lines of treatment) | 6  2 | 6  2 | 4  1 |  | 6 | 2 |
| Ibrutinib (n=1)  (R/R after 2 lines of treatment) | 1 | 1 | 0 |  | 1 |  |

SUPPLEMENTAL TABLE S4. del(11q) clonal progression

*del(11q) was no longer detected after FCR (CLL_1)

FCR: fludarabine/cyclophosphamide/rituximab; BR: bendamustine, rituximab; BOMP: bendamustine, ofatumumab, methylprednisolone
